# Water Status Related Root-to-Shoot Communication Regulates the Chilling Tolerance of Shoot in Cucumber (*Cucumis sativus* L.) Plants

**DOI:** 10.1038/srep13094

**Published:** 2015-10-16

**Authors:** Zi-Shan Zhang, Mei-Jun Liu, Hui-Yuan Gao, Li-Qiao Jin, Yu-Ting Li, Qing-Ming Li, Xi-Zhen Ai

**Affiliations:** 1 State Key Lab of Crop Biology, Tai’an, Shandong Province, China; 2 College of Life Sciences, Shandong Agricultural University, Tai’an, Shandong Province, China; 3 College of Horticulture Science and Engineering, Shandong Agricultural University, Tai’an, Shandong Province, China

## Abstract

Although root-to-shoot communication has been intensively investigated in plants under drought, few studies have examined root-to-shoot communication under chilling. Here we explored whether root-to-shoot communication contributes to the chilling-light tolerance of cucumber shoots and clarified the key signal involves in this communication. After leaf discs chilling-light treatment, the photoinhibitions of Photosystem I (PSI) and Photosystem II (PSII) were similar in leaf discs of two cucumber varieties (JY-3 and JC-4). When the whole plants, including roots, were chilled under light, the photosynthetic performances in JC-4 leaves decreased more seriously than that in JY-3 leaves. However, when the water status of leaves was maintained by warming roots or floating the attached leaves on water, the PSII activity and amount of PSI in the leaves of the two varieties were similar after chilling-light treatment. In addition, the differences of PSII activities and amount of PSI between the two varieties under whole plant chilling-light treatment were independent of ABA pretreatment. Above results indicate that (1) the better water status in leaves under chilling contributes to the higher chilling tolerance of JY-3; (2) the water status, rather than an ABA signal, dominates root-to-shoot communication under chilling and the chilling tolerance of cucumber shoot.

In higher plants, organs that contribute to the organization of the entire plant have different functions, and the growth of these organs depends on the interactions among them. Numerous studies have shown that plant roots can sense changes in abiotic factors such as water content^1^,^2^, oxygen content^3^, and the nutrient composition^4^ of the soil. Roots form signals that transported though xylem to shoots and affected the shoot physiological metabolism^5^.

Two possibilities exist for long-distance signaling in root-to-shoot communication: chemical signals and water status which was also called as hydraulic signal^5^,^6^. The former class includes abscisic acid (ABA), pH, cytokinins, ethylene precursors, malate and other unidentified factors^5^. Among these chemical signals, ABA attracts the most attention^2^,^7^,^8^. According to current understanding, under drought conditions, roots sense the soil water shortage and produce or release ABA which is then transported through the xylem to the shoot; the guard cells respond to the increase of ABA content by reducing stomatal conductance (Gs), which is advantageous for decreasing transpiration rate and increasing drought tolerance of plant^2^.

Although the root-to-shoot communication have been intensively investigated in plants exposed to drought^6^,^8^,^9^,^10^,^11^,^12^, few studies have been conducted on root-to-shoot communication in plants under chilling stress. It has been reported that the chilling exposure of roots decreased the root hydraulic conductivity, root pressure, xylem sap transport, as well as the activities of plasma membrane H^+^-ATPase and aquaporin^13^,^14^,^15^,^16^,^17^,^18^,^19^. Most of previous researches about root chilling focus the effects of root chilling and shoot illumination on the hydraulic properties of root^18^,^20^, however, it has not been clarified that whether the response of root to chilling contributes to the chilling tolerance of shoot. It recently has been shown that compared with whole plant chilling-light treatment (L/L), severer injury of leaves was induced by the treatment where only leaves but not roots were chilled (L/H) in some chilling-sensitive species, such as *Oryza sativa*^21^,^22^ and *Spathiphyllum wallisii*^23^. The chilling injury of leaves induced by L/H is related to the malfunction of chlororespiration^24^ and excess supply of water and nutrition^22^,^25^.

Cucumber (*Cucumis sativus* L.) is a typically chilling-sensitive species and is one of the most important vegetable species in protected cultivation. Cucumber plants commonly encounter chilling stress in unheated greenhouses^26^,^27^. To enhance the chilling tolerance of cucumber plants, farmers have traditionally grafted cucumber shoots onto rootstocks from other more chilling-tolerant species such as the figleaf gourd. The studies of Zhou *et al.*^26^,^27^ demonstrated that, compared with cucumber plants using their own roots, plants grafted onto figleaf gourd roots have higher photosynthetic rate, Gs and lower photoinhibition under chilling-light treatment (14 °C or 7 °C, 100 μmol m^−2^ s^−1^). However, it remains unclear how a more chilling-tolerant root contributes to the improvement of photosynthetic performance in shoots under chilling-light conditions, and the signals involved in root-to-shoot communication under chilling stress are also unknown. To address these questions, we examined the phenotypic change, water status, gas exchange, the activity of photosystem II (PSII) and the amount of photosystem I (PSI) in leaves of the chilling-sensitive cucumber variety Jinchun No. 4 (JC-4) and the chilling-tolerant cucumber variety Jinyou No. 3 (JY-3) under different chilling treatments.

## Results

### Photosynthetic performance in the detached leaf discs treated with cool water under light

**Figure 1:**
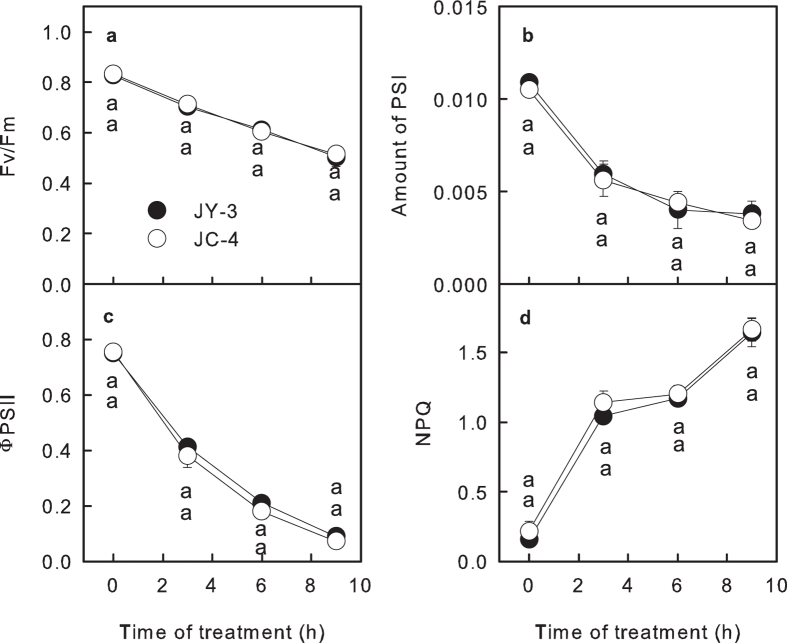
The tolerances of JY-3 and JC-4 when the detached leaf discs were treated with cool water under light. The changes of the maximum quantum yield of PSII (Fv/Fm, **a**) the amount of PSI (**b**) the quantum yield of electron transfer through PSII (ΦPSII, **c**) and the non-photochemical quenching (NPQ, **d**) in detached leaf discs of JY-3 and JC-4 cucumber under chilling-light treatment for different periods of time. Leaf discs punched from fully expanded leaves were floated on the surface of cool water (6 °C), and the light intensity on the surface of the leaf discs was 150 μmol m^−2^ s^−1^. Values represent means ± SE, and each data point is the average of 10 independent plants. Different letters indicate significant differences between different cucumber varieties at P < 0.05.

Leaf discs punched from fully expanded leaves were floated on the surface of cool water (6 °C) under 150 μmol m^−2^ s^−1^ light. The maximum quantum yield of PSII (Fv/Fm), the quantum yield of electron transfer through PSII (ΦPSII) and the amount of PSI in the leaf discs obviously decreased, and the non-photochemical quenching (NPQ) increased after chilling-light treatment (Fig. 1), indicating that the chilling-light treatment resulted in PSII and PSI photoinhibitions and induced photoprotection mechanism. However, no significant differences in Fv/Fm, ΦPSII, NPQ and the amount of PSI were observed between the leaf discs from the JC-4 and those from the JY-3 during the 9 h period of chilling-light treatment (Fig. 1), indicating that the leaf discs from the JC-4 actually had a similar chilling tolerance to the leaf discs from the JY-3.

### Changes of photosynthetic performance in the leaves of cucumber under whole plant chilling-light treatment

**Figure 2:**
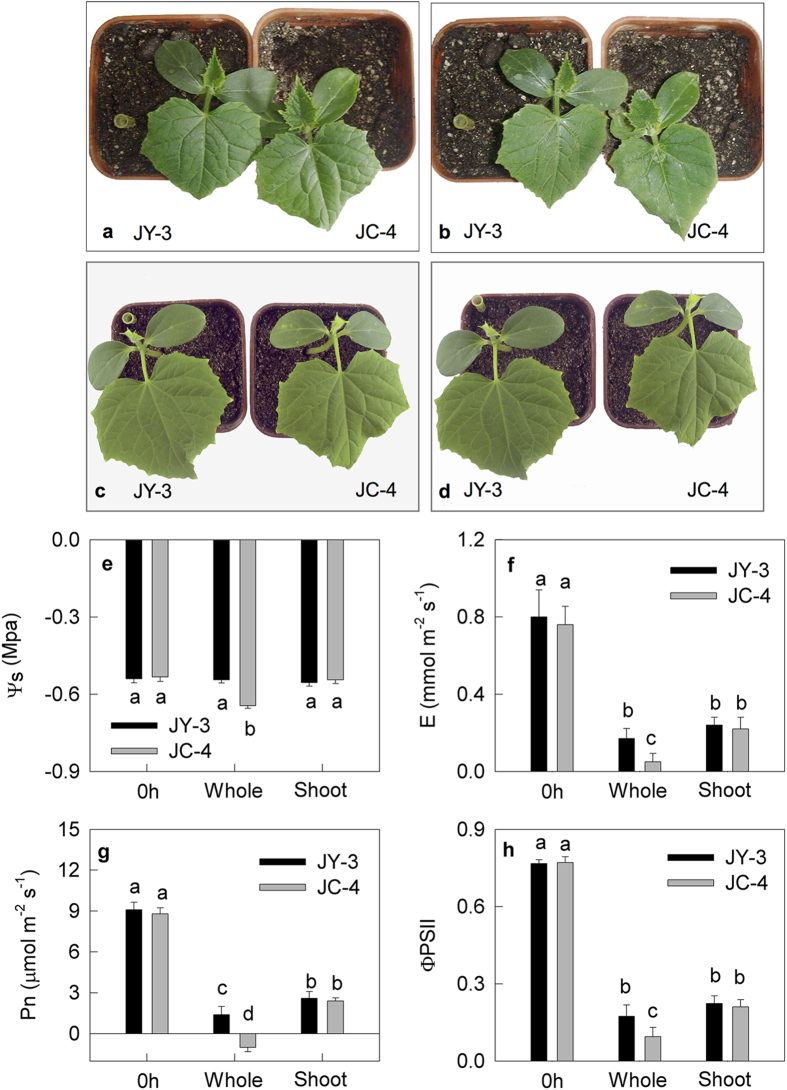
The characterization of leaves of JY-3 and JC-4 under whole plant (including the root) or shoot-special chilling-light treatment. The phenotypes of the chilling-tolerant cucumber variety JY-3 and the chilling-sensitive cucumber variety JC-4 before (**a**,**c**) and after (**b**) 9 h of whole plant chilling-light treatment or shoot-special chilling-light treatment (**d**) The osmotic potential (Ψ_s_, **e**) transpiration rate (E, **f**) net photosynthetic rate (Pn, **g**) the quantum yield of electron transfer through PSII (ΦPSII, **h**) in the JY-3 leaves and the JC-4 leaves before and after 9 h of whole plant chilling-light treatment (whole) or shoot-special chilling-light treatment (shoot; 6 °C and 150 μmol m^−2^ s^−1^ light). Values represent means ± SE, and each data point is the average of 10 independent plants. Different letters indicate significant differences at P < 0.05.

After the whole plants, including the roots, were treated at 6 °C under 150 μmol m^−2^ s^−1^ light for 9 h, obvious wilting and a significant decrease in osmotic potential (Ψ_s_) were observed in JC-4 leaves, but not in JY-3 leaves (Fig. 2a,b,e). The decreases of transpiration rate (E), net photosynthetic rate (Pn) and ΦPSII were more obvious in JC-4 leaves than in JY-3 leaves after 9 h of chilling-light treatment (Fig. 2f–h).

**Figure 3:**
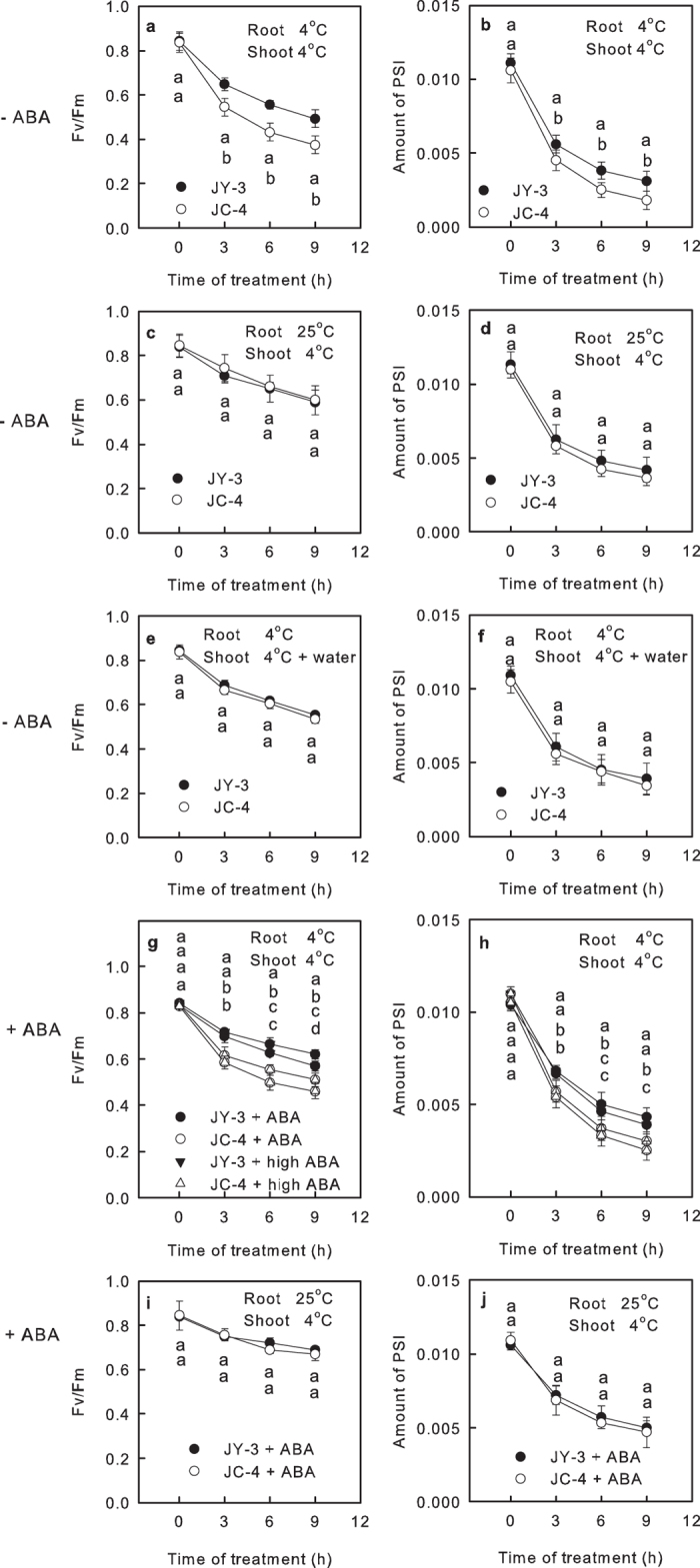
Effects of leaf water status and ABA signal on the chilling tolerance of JY-3 leaves and JC-4 leaves. The maximum quantum yield of PSII (Fv/Fm, **a**,**c**,**e**,**g**,**i**) and the amount of PSI (**b**,**d**,**f**,**h**,**j**) in leaves of JY-3 and JC-4 seedings that exposed to whole plant chilling-light treatment (**a**,**b**,**g**,**h**), shoot-special chilling-light treatment (**c**,**d**,**i**,**j**) and whole plant chilling-light treatment with attached leaves floated on cool water (**e**,**f**) under 150 μmol m^−2^ s^−1^ light. Before chilling-light treatment, the leaves were sprayed by 100 μM (+ABA) or 1000 μM (+high ABA) ABA solution (with 0.02% Tween 20) or 0.02% Tween 20 solution (-ABA). In the whole plant chilling treatment, plants were exposed to 6 °C. In the shoot-special chilling treatment, the shoots were exposed to 6 °C and the roots were exposed to 25  °C. Values represent the means ± SE, and each data point is the average of 10 independent plants. Different letters indicate significant differences between different cucumber varieties at P < 0.05.

We also analyzed the photoinhibition of PSII and PSI in leaves during whole plant chilling-light treatment. It was observed that the Fv/Fm and the amount of PSI were similar in leaves of the two cucumber varieties under growth temperature (25 °C; Fig. 3a,b). After 9 h whole plant chilling-light treatment, the Fv/Fm and the amount of PSI decreased more seriously in JC-4 leaves than in JY-3 leaves (Fig. 3a,b), indicating that both the PSII and PSI was damaged more severely in JC-4 leaves than JY-3 leaves during chilling-light treatment.

The above result demonstrates that when the whole plants of the two varieties were treated with chilling-light, the JY-3 was more chilling-tolerant than the JC-4, which was inconsistent with the results obtained from the detached leaf discs experiment. We speculated that the root of the cucumber seeding and the root-to-shoot communication play an important role in the chilling tolerance of the shoot. To investigate this hypothesis, we exposed the shoots and roots of cucumber seedlings to different temperatures.

### Changes of photosynthetic performance in the leaves of cucumber seedlings under shoot-special chilling-light treatment

After the shoots were exposed to chilling-light condition (6 °C and 150 μmol m^−2^ s^−1^ light) and the roots were maintained at 25 °C simultaneously for 9 h, no wilting was observed in either the JY-3 leaves or the JC-4 leaves (Fig. 2c,d). The Ψ_s_ of leaves were not significantly changed in the two varieties (Fig. 2e). The E, Pn and ΦPSII significantly decreased in the leaves of both varieties after shoot-special chilling-light treatment, but these decreases were similar in the two varieties (Fig. 2f–h). No significant differences were observed in the Fv/Fm and the amount of PSI between the JY-3 leaves and the JC-4 leaves, although the Fv/Fm and the amount of PSI decreased remarkably in leaves of the two varieties after shoot-special chilling-light treatment (Fig. 3c,d). These results support the hypothesis that the root of the cucumber and the root-to-shoot communication play an important role in the chilling tolerance of the shoot.

Two kinds of long-distance signals are possible in root-to-shoot communication: chemical signals and water status^5^,^6^. The most representative chemical signal is ABA^2^,^7^,^8^. To further investigate whether a chemical signal (ABA) or water status participates in root-to-shoot communication under chilling, we manipulated the water status and the ABA level in leaves and analyzed the effects of the water status and the ABA level on the PSI and PSII photoinhibition of leaves under chilling-light treatment in further experiments.

### Effects of leaf water status and ABA signal on the chilling tolerance of cucumber leaves

When the shoots of the plants were treated with chilling-light and the roots were exposed to 25 °C (Fig. 3c,d), or when the whole plants were treated with chilling temperatures and their attached leaves were floated on cool water (6 °C; Fig. 3e,f), no significant differences were observed in the Fv/Fm and the amount of PSI in the JY-3 leaves and the JC-4 leaves. These results demonstrate that as long as the leaf water status was maintained during the chilling treatment, the difference of chilling sensitivity between the two cucumber varieties was nonexistent.

In order to examine the effect of ABA content to the difference of chilling-light tolerance between JC-4 and JY-3 leaves, we sprayed ABA solution on the leaves before chilling-light treatment. The Gs of leaves under light steeply decreased after ABA pretreatment (Supplementary Fig. S1), which proves that the ABA pretreatment increased the ABA content in leaves. The pretreatment of 100 μM ABA solution (a commonly concentration of ABA used in physiology and biochemistry studies^28^,^29^,^30^) alleviated the decreases of Fv/Fm and the amount of PSI during the whole plant chilling-light treatment (Fig. 3g,h). The pretreatment with an extra-high concentration of ABA (1000 μM) led to similar results (Fig. 3g,h). The decrease of Fv/Fm in 1000 μM ABA treated leaves was slightly severer than that in 100 μM ABA treated leaves, which indicates that high concentration of ABA had weak side effects on Fv/Fm. The potential difference of ABA content between the leaves of two varieties can be eliminated by the extra-high concentration of ABA, however, the differences of Fv/Fm and the amount of PSI between the two cucumber varieties after whole-plant or shoot-special chilling treatment were independent of ABA pretreatment (Fig. 3g–j). This result showed that although ABA pretreatment increased the chilling-light tolerance of cucumber leaves, the responses to the ABA in the two cucumber varieties were similar; the difference in chilling-light tolerant between the two cucumber varieties was independent of the ABA signal.

### Effects of root chilling on root-to-shoot communication

Stomatal closure is a model phenomenon for determining increase of effective ABA content in leaves^9^,^31^. To further verify whether ABA participates in root-to-shoot communication under chilling, we detected the gas exchange of leaves at 25 °C during the roots was exposed to chilling shock (6 °C).

**Figure 4:**
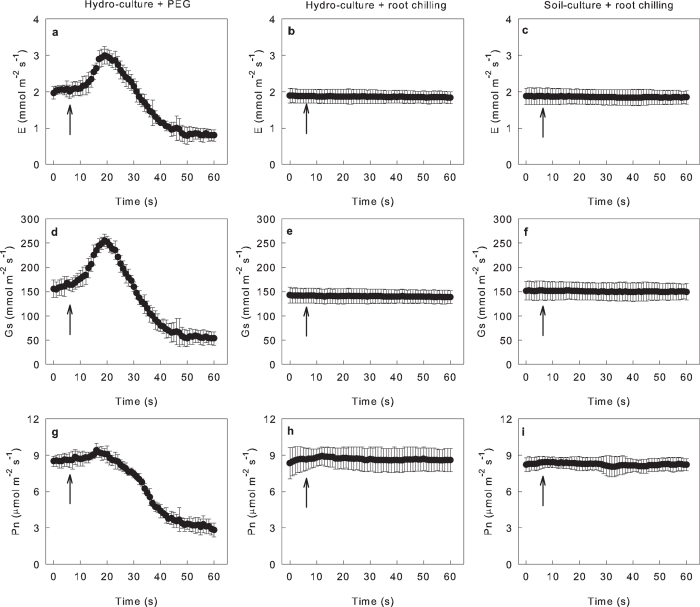
Effects of root chilling on the photosynthetic gas exchange of leaves. The responses of transpiration rate (E; **a**–**c**), stomatal conductance (Gs; **d**–**f**) and net photosynthetic rate (Pn; **g**–**i**) in JC-4 leaves to PEG (polyethylene glycol, 30%, W/W) shock at 25 °C (**a**,**d**,**g**) and chilling shock (6 °C; **b**,**c**,**e**,**f**,**h**,**i**) of root. The plants were grown in Hoagland nutrient solution (**a**,**b**,**d**,**e**,**g**,**h**) or soil (**c**,**f**,**i**). The arrowheads indicate the start of shock. The gas exchange in the leaves was measured at 800 μmol mol^−1^s light, 25 °C, 400 μmol mol^−1^ CO_2_ concentration and 60–65% relative humidity. Each data point is the average of 4 independent plants. The results for the chilling-tolerant cucumber variety JY-3 were similar to those of the JC-4 (Supplementary Fig. S2).

As shown in Fig. 4, PEG-induced (polyethylene glycol) drought shock induced severe fluctuations and decreases in E, Gs and Pn; however, root chilling shock did not influence the gas exchange in JC-4 leaves (Fig. 4) and JY-3 leaves (Supplementary Fig. S2). The above results demonstrate that when the root senses chilling, it does not transport ABA or another chemical to the shoot to increase the effective ABA content in the leaves.

## Discussion

This study provided evidences demonstrating that, compared to the JC-4 cucumber, the higher chilling-light tolerance of the JY-3 cucumber was caused by the better water status in JY-3 leaves. Water status but not ABA, dominates root-to-shoot communication under chilling and the chilling tolerance of cucumber leaves.

Previous studies have intensively focused on root-to-shoot communication under drought conditions^6^,^12^,^32^,^33^,^34^; however, few studies have been conducted on root-to-shoot communication under chilling condition. Similarly to drought, chilling also induces shoot water deficit (Fig. 2). This water deficit is induced by the reduced water-absorbing capacity of the root due to the suppression of mitochondrial respiration, H^+^-ATPase and aquaporin activities in the root under chilling^14^,^15^,^35^. However, the effects of drought and chilling to plant are different: chilling not only affects the shoot directly but also affects it indirectly through root-to-shoot interactions, while drought only affects the shoot indirectly through root-to-shoot interactions. Therefore, the study of root-to-shoot communication under chilling conditions is more complex than that under drought conditions. In this study, we controlled the temperature of root and shoot respectively to examine root-to-shoot communication specifically under chilling conditions. Chilling treatment inhibits the activity of enzymes related to the Calvin cycle and therefore sharply suppresses photosynthesis; the inhibition of photosynthesis inevitably causes stomatal closure, so we cannot estimate root-to-shoot communication under chilling conditions through the analysis of stomata behavior, although the stomatal behavior has been widely used to estimate root-to-shoot communication under drought conditions^6^,^9^,^12^,^31^. For example, we observed in this study that when the whole plants were exposed to chilling, the Pn and Gs in the chilling-tolerant variety JY-3 were much higher than that in the chilling-sensitive variety JC-4 (Fig. 2), however, the two varieties had similar substomatal CO_2_ concentrations (Ci, Supplementary Fig. S3), indicating that the difference of Pn between the two cucumber varieties was primarily caused by the different carbon fixation capabilities of their mesophyll cells rather than stomatal factors. Therefore, in order to estimate the root-to-shoot communication under chilling conditions, we must globally analyze the physiological state of the leaves, particularly the photosynthetic performance, which is one of the most sensitive factors to the environment. It has been reported that the PSI is more sensitive to chilling-light stress than the PSII^36^,^37^,^38^, and the recovery of PSI is much slower than PSII after stress^37^,^39^,^40^, so the PSI photoinhibition should be paid more attention to. This study indicates that the integrative analysis of gas exchange and the activity of the two photosystems is an effective tool to estimate the occurrence of root-to-shoot communication under chilling-light conditions.

Zhou *et al.* reported that compared with cucumber plants grafted onto the roots of figleaf gourd, cucumber plants with their own roots had more severe suppression of photosynthetic performance under chilling-light condition, accompanied by obviously different hormone concentrations in their root xylem exudates^21^. However, these parallel results are insufficient to prove that the hormone transport from root to shoot dominates the chilling tolerance of photosynthetic performance in shoot. This study provided evidence to demonstrate that the water status, but not ABA signals, dominates root-to-shoot communication and the chilling tolerance of shoots under chilling-light treatment. This conclusion was supported by the following results: first, this study showed that the difference of shoot chilling tolerance between the two cucumber varieties was nonexistent when the leaf water state was maintained by warming the roots or floating the attached leaves on water, regardless of root temperature (Figs 2 and 3). This fact indicates that the chilling-light tolerance of cucumber shoot was dependent on the water state in leaves under chilling, and the hydraulic signal dominates the root-to-shoot communication in cucumber seeding under chilling.

Second, we pretreated the leaves with extra-high concentration of ABA to eliminate the potential difference of ABA content between the leaves of two varieties, but the ABA pretreatment did not affect the difference of shoot chilling tolerance between the two cucumber varieties (Fig. 3), which indicating that the difference of chilling tolerance between the two cucumber varieties was independent of the ABA content of leaves.

Third, the chilling shock of root did not induce stomatal closure in leaves (Fig. 4, S2), indicating that when the root senses chilling, it does not transport ABA or another chemical to the shoot to increase the effective ABA content in the leaves, which is different with the root-shoot communication under drought stress. It was reported that hydraulic flux of root decreased immediately upon exposure of roots to chilling^41^, indicating that the hydraulic signal respond to the root-chilling treatment much faster than ABA signal. Wan *et al.* reported that the lowest concentration of exogenous ABA to induce stomatal closure was several-fold higher compared with the concentration present in the xylem sap of aspen under root chilling^42^. The transpiration rate of leaves was severely inhibited under chilling, so the ABA in xylem sap cannot be concentrated by water loss in leaves. Therefore, the stomatal closure in leaves under chilling was not caused by the ABA from roots. Together with our results, we suggest that the water status, rather than ABA, contributes to root-to-shoot communication and dominates the chilling tolerance of shoots under chilling-light treatment.

It should be noted that possible long-distance chemical signals for root-to-shoot communication other than ABA include pH, cytokinins, ethylene precursors, malate and other unidentified factors^5^, this study did not analyze all of them but only ABA due to the huge workload. However, the fact that the difference of shoot chilling-light tolerance between the two cucumber varieties disappeared when the leaf water state was maintained during chilling-light treatment strongly supports the conclusion that the water status dominates root-to-shoot communication in cucumber plants. However, this study cannot exclude the possibility that chemical signals contribute to root-to-shoot communication and regulate the chilling tolerance of cucumber leaves at the downstream of the water status in shoot. More studies are needed to clarify the signal pathway of root-to-shoot communication under chilling.

In conclusion, this study demonstrated that, compared to the JC-4, the higher chilling tolerance of the JY-3 leaves is caused by the better water status of JY-3 leaves; the water status, but not chemical signals such as ABA, dominates root-to-shoot communication under chilling and regulated the chilling tolerance of shoots.

## Materials and Methods

### Plant material

Two cucumber varieties, Jinchun No. 4 (JC-4) and Jinyou No. 3 (JY-3), were used in this study. Before this study we comparatively studied the chilling sensitivity of the two cucumber variety to confirm that the leaves of JC-4 are more chilling sensitive than that of JY-3 in field. So in this study, we used JC-4 as chilling-sensitive variety and JY-3 as chilling-tolerant variety. The plants were planted in pots (7 cm in diameter, 10 cm in height) filled with rich soil or Hoagland nutrient solution. Rich soil could supply sufficient nutrients to plants. Enough water was supplied to the plants grown in soil. Nutrient solution was renewed everyday to avoid any potential nutrient deficiency. The plants were grew in a growth chamber at 25 °C/22 °C and 150 μmol m^−2^ s^−1^ with a 14 h/10 h photoperiod. Plants were used for experiments when their first true leaves had fully expanded. The plants grown in nutrient solution were only used in the photosynthesis analysis when the roots were shocked by PEG (polyethylene glycol) or chilling.

### Chilling-light treatment

In the detached leaf treatment, leaf discs (1 cm^2^) were punched from the fully expanded leaves and floated on the surface of water at 6 °C. The temperature was controlled using a GXZ-5000 incubator (Jiangnan, China).

In the whole plant treatment, the plants were placed in a GXZ-5000 incubator, with the containers of the roots being placed in a water bath connected to an RTE-211 water circulator (NESLAB, USA). The shoot temperature was controlled by GXZ-5000 incubator, the root temperature was controlled by RTE-211 water circulator. Three different temperature treatments were performed in this study: 1, the whole plants were exposed to 6 °C; 2, the shoots were exposed to 6 °C but the roots were exposed to 25 °C; and 3, the shoots were exposed to 25 °C but the roots were exposed to 6 °C.

A red and blue light emitting diode (LED) light source (SP501-N, SanPeng, China) was used to provide illumination, the light intensity on the surfaces of the leaf discs or the attached leaves was maintained at 150 μmol m^−2^ s^−1^.

### ABA treatment

Either 100 or 1000 μM abscisic acid solution (ABA; Sigma, USA) with 0.02% Tween 20 was sprayed on the leaves three times at 20 min intervals. The control solution was 0.02% Tween 20. The following experiments were initiated 1 h after the ABA treatment.

### Measurements of chlorophyll fluorescence and the changes in absorbance at 820 nm

The maximum quantum yield of PSII (Fv/Fm) were measured using a Handy PEA fluorometer (Hansatech, UK) on leaves that had been dark-adapted under 25 °C for 30 min. A saturating red light of 3000 μmol m^−2^ s^−1^ was produced by an array of four LEDs (peak 650 nm).

Modulated chlorophyll fluorescence was measured with a FMS-2 pulse-modulated fluorometer (Hansatech, UK). The measurement protocol is as follows: dark-adapted leaves were irradiated by a 0.8 s saturating light of 8000 μmol m^−2^ s^−1^ to obtain maximum fluorescence in the dark-adapted state (Fm); the light-adapted leaves were continuously illuminated by an actinic light of 150 μmol m^−2^ s^−1^ from the FMS-2 light source, and steady-state fluorescence (Fs’) was recorded after 2 min of illumination. Finally, a 0.8 s saturating light of 8000 μmol m^−2^ s^−1^ was imposed to obtain the maximum fluorescence in the light-adapted state (Fm’).

The following parameters were calculated^43^: (1) maximum quantum yield of PSII, (Fv/Fm) = 1 - (Fo/Fm); (2) quantum yield of electron transfer through PSII, ΦPSII = (Fm’ − Fs’)/Fm’; (3) non-photochemical quenching, NPQ = (Fm − Fm’)/Fm’.

The changes in absorbance at 820 nm were measured with a M-PEA fluorometer (Hansatech, UK) according to Zhang *et al.*^38^,^40^. A dark-adapted leaf was illuminated with far-red light (250 μmol m^−2^ s^−1^) at time 0 s, until a steady P700 photooxidation was reached, then the far-red light was closed (at time 20 s). A modulated (33.3 kHz) measuring light at 820 nm was provided simultaneously with the illumination of far-red light. The change in the amplitude of 820 nm was used to compare the amount of PSI.

### Photosynthetic gas exchange measurements

The net photosynthetic rate (Pn), stomatal conductance (Gs), transpiration rate (E) and substomatal CO_2_ concentrations (Ci) were measured using a CIRAS-2 portable photosynthesis system (PP Systems, USA) at 6 °C or 25 °C, 150 μmol m^−2^ s^−1^ light, 400 μmol mol^−1^ CO_2_ and 60–65% relative humidity. Leaf temperature, light intensity, CO_2_ concentration and relative humidity were controlled using the automatic control device of the CIRAS-2 portable photosynthetic system. To prove that if the pretreatment with ABA solution increased the ABA content in leaves, the Gs of leaves were measured under 800 μmol mol^−1^s light.

To analyze the response of photosynthesis to the PEG shock and chilling shock of root, the plants grown in Hoagland nutrient solution and the plants grown in soil were used. Attached leaves were irradiated by 800 μmol mol^−1^s light supplied by CIRAS-2 under 25 °C, 400 μmol mol^−1^ CO_2_ concentration and 60–65% relative humidity. After steady-state photosynthesis was reached, the Hoagland solution was rapidly replaced by Hoagland solution contain 30% (W/W) PEG 6000 at 25 °C or cool Hoagland solution (6 °C). The Pn, Gs and E were automatically recorded every 60 s by CIRAS-2. During measurements, the roots were placed in a water bath connected to an RTE-211 water circulator, the temperature of roots were controlled by RTE-211 water circulator. The air temperature were maintain at 25 °C by GXZ-5000 light incubator and CIRAS-2 portable photosynthesis system.

### Determination of water status

Leaf osmotic potential (Ψ_s_) was measured using a 5520 Vapor Pressure Osmometer (Wescor, USA) after the cell sap was collected, according to the methods of Bajji *et al.*^44^.

### Statistical analyses

Independent measurements were done in 10 independent plants for all experiments expect that the PEG and chilling shock experiments were done in 4 independent plants. The calculations for standard error (SE) were conducted using Microsoft Excel software. LSD (least significant difference) tests were conducted to analyze the differences between varieties using SPSS 16.

## Additional Information

**How to cite this article**: Zhang, Z.-S. *et al.* Water Status Related Root-to-Shoot Communication Regulates the Chilling Tolerance of Shoot in Cucumber (*Cucumis sativus* L.) Plants. *Sci. Rep.* **5**, 13094; doi: 10.1038/srep13094 (2015).

## Supplementary Material

Supplementary Information
