# Supplementary material for: Water Status Related Root-to-Shoot Communication Regulates the Chilling Tolerance of Shoot in Cucumber (Cucumis sativus L.) Plants
Source: Sci Rep. 2015 Oct 16;5:13094. doi: 10.1038/srep13094 (PMC4607976; doi:10.1038/srep13094)

Title: Water Status Related Root-to-Shoot Communication Regulates the Chilling Tolerance of Shoot in Cucumber (*Cucumis sativus* L.) Plants

Zi-Shan Zhang[1,2,3], Mei-Jun Liu[1,2], Hui-Yuan Gao[1,2*], Li-Qiao Jin[1,2], Yu-Ting Li[1,2], Qing-Ming Li[2,3], Xi-Zhen Ai[2,3]

[1]State Key Lab of Crop Biology, Tai'an, Shandong Province, China, [2]College of Life Sciences, Shandong Agricultural University, Tai'an, Shandong Province, China, [3]College of Horticulture Science and Engineering, Shandong Agricultural University, Tai'an, Shandong Province, China

[*]Corresponding author: Hui-Yuan Gao; E-mail: gaohy@sdau.edu.cn

Supplementary Figure. S1 The stomatal conductance (Gs) under light (800 μmol m^{-1} s^{-1}) and 25 °C in JC-4 and JY-3 leaves pretreated with 0, 100 or 1000 μM ABA solution.

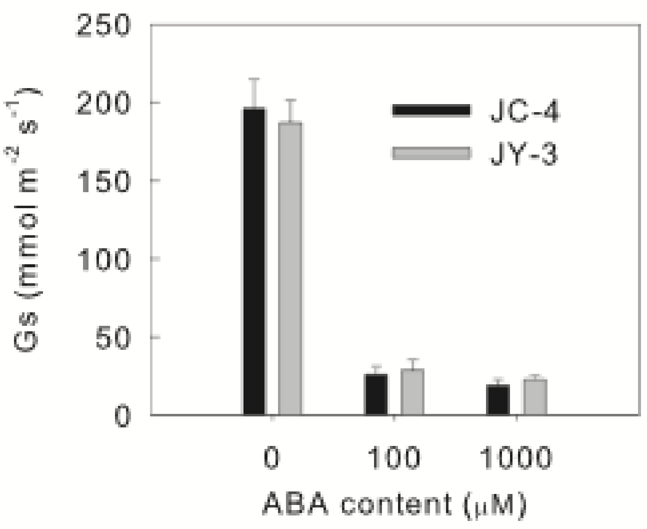

Supplementary Figure S2: The responses of transpiration rate (E; a, b, c), stomatal conductance (Gs; d, e, f) and net photosynthetic rate (Pn; g, h, i) in leaves of the chilling-sensitive cucumber variety JY-3 to PEG (polyethylene glycol, 30%, W/W) shock (a, d, g) and chilling shock (6 ^{o}C; b, c, e, f, g, i) of root. The plants were grown in Hoagland nutrient solution (a, b, d, e, g, h) or soil (c, f, i). The arrowheads indicate the start of shock. The gas exchange in the leaves was measured at 800 μmol mol^{-1} s^{-1} light, 25 °C, 400 μmol mol^{-1} CO_2 concentration and about 65% relative humidity. The results for the chilling-tolerant cucumber variety JC-4 were similar to those of the JY-3 (Fig. 5).

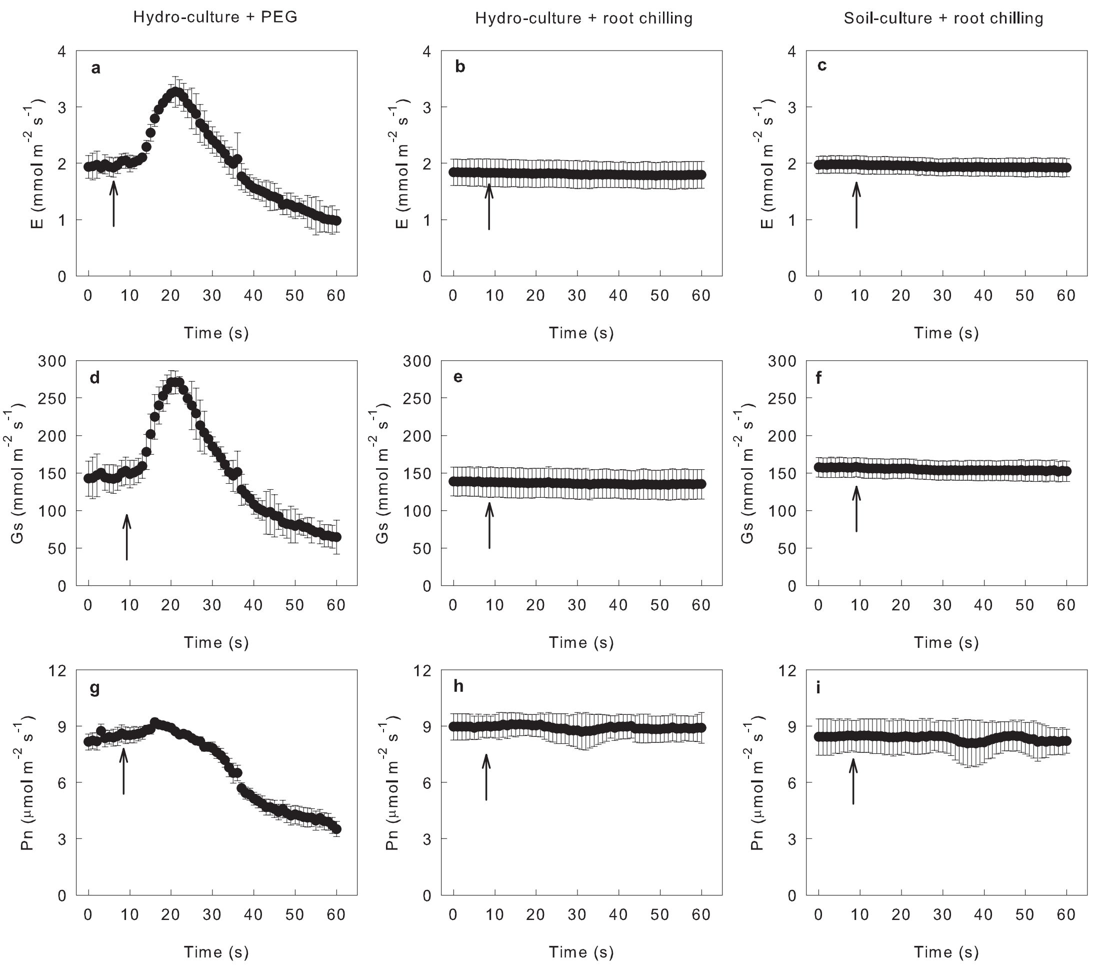

Supplementary Figure S3: The substomatal CO_2 concentrations (Ci) in the JY-3 leaves and the JC-4 leaves before and after 9 h of whole plants chilling-light treatment (6°C and 150 μmol m^{-2} s^{-1} light). Values represent means ± SD, and each data point is the average of 10 independent plants.

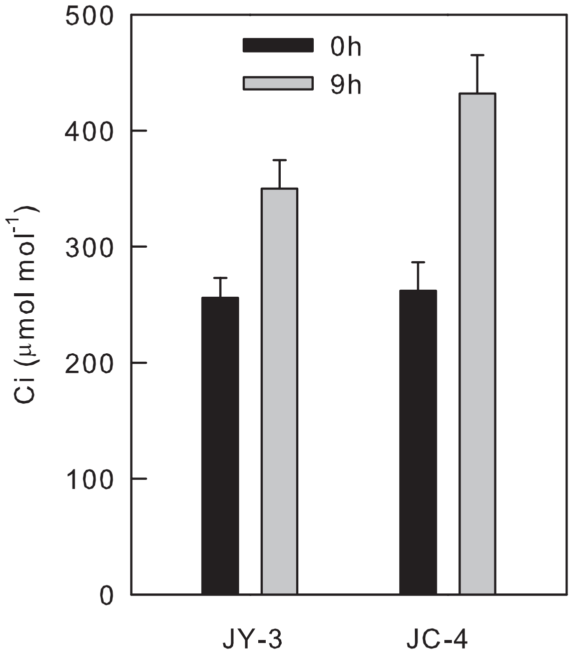

Supplement: Supplementary Information [file srep13094-s1.pdf]
